# Editorial: Immune Response to Cerebral Ischemia: Exploring Mechanisms and Potential Treatment Targets

**DOI:** 10.3389/fimmu.2021.813836

**Published:** 2021-12-24

**Authors:** YingZe Ye, Lijuan Gu, Zhihong Jian, Xiaoxing Xiong

**Affiliations:** ^1^ Central Laboratory, Renmin Hospital of Wuhan University, Wuhan, China; ^2^ Department of Neurosurgery, Renmin Hospital of Wuhan University, Wuhan, China

**Keywords:** cerebral ischemia, neuroinflammation, peripheral immune system, immune cells, immune mediators, immunotherapy

Cerebral ischemia or ischemic stroke (IS) is a leading cause of death worldwide, only behind cardiac ischemia. However, the only effective medication currently approved by the Food and Drug Administration for the treatment of acute ischemic stroke is the thrombolytic tissue plasminogen activator. Therefore, to further clarify the intricate mechanisms so as to explore novel therapeutic strategies is pretty critical. Immunity and inflammation, whether at the acute stage shown as arterial occlusion-initiated brain damage or in the late phase presented with tissue repair, are instrumental and indispensable in the ischemic cascade. Recently, research developments have unveiled that the brain and the immune system have a bidirectional cross-talk, featured by the infiltration of peripheral immune cells in the damaged brain due to the blood–brain barrier (BBB) disruption after IS has occurred, stroke-induced immunosuppression, and subsequent infection, which enormously challenge stroke therapy. Studies in rodent models suggest that the modulation of post-ischemic immunity and inflammation, terribly important links of the ischemic cascade, offers unique benefits. However, immunoregulation is not devoid of deleterious side effects, especially the aggravation of immunodepression. Another issue is that restraining the inflammatory response may remit the cerebral injury in the early phase but imperil the brain through destructing repair mechanisms, thus worsening the long-term outcome. The essential and inconsistent role of inflammation at different stages of stroke brings concern as to whether methods based on the complete suppression of inflammation are feasible. Therefore, a more comprehensive understanding of stroke-induced immunology and inflammation will enable the development of selective and purposeful approaches to suppress their destructive effects.

The Research Topic *Immune Response to Cerebral Ischemia: Exploring Mechanisms and Potential Treatment Targets* focuses on the immunological mechanisms of cerebral ischemia, aiming to explore the interaction between the immune system and ischemic stroke and to discuss and speculate the treatment landscape targeting immunity.

Ge Gao et al. observed that glutaminase 1 (GLS1), a primary enzyme responsible for the generation of glutamate in the central nervous system, was increased in rat brains at 72 h post-focal cerebral ischemia, accompanied by pro-inflammatory exosome release and microglia activation, while glutaminase inhibitor suppressed microglial activation, exosome secretion, and neuroinflammation, which suggests that GLS1 mediates exosome release and neuroinflammatory microenvironment formation as synergistically demonstrated through the application of an exosome inhibitor. Zhili Chen et al. reported that stroke resulted in the production of brain-derived microparticles (BDMP), and BDMP treatment in stroke mice increased the infarct volume, aggravated the BBB leakage, and enhanced the microglial activation and inflammatory cell infiltration as well as pro-inflammatory factor expression. The BDMP scavenger improved the above-mentioned neurological outcomes and mitigated neuroinflammation. Ting Liu et al. found that proinflammatory cytokine interleukin 17A aggravated the OGD/R-induced ischemic injuries of primary neurons by promoting the initiation of an excessive autophagic process. In summary, treatments targeting the regulation of inflammatory mediators are expected to enhance the curative effect and will be an essential direction in IS treatment.

It is well known that immune cells play very important roles in IS. Zhihong Jian et al. reviewed the different timelines and roles of infiltrated residential and peripheral immune cells in the brain at the distinctive stages of stroke. They emphasized the sources, phenotype transformation, and function alteration of microglia-derived macrophages and monocyte-derived macrophages during cerebral ischemia. Yijie Wang et al. also discussed the phenotypes, functions, released mediators, and upstream regulatory agents of different subpopulations of both cerebral and peripheral immune cells as well as their cross-talk after IS occurrence. Targeting distinctive immune cells and specifically regulating their function may hereby be a novel idea for inhibiting neuroinflammatory injury and promoting neural restoration at different time courses of IS.

Immunity and inflammation actively modulate the pathophysiological processes of IS; the injured brain also communicates with the immune system and peripheral organs. Ying Kong et al. collected peripheral blood natural killer (NK) cells from patients with acute ischemic stroke, measured them, and found that the number of NK cells was reduced, the immune phenotyping was changed, and the extent in number depletion and activity alteration was associated with brain infarct size. Fangxi Liu et al. reviewed the cross-talk between the brain and the immune system after stroke and pointed out that the beneficial or detrimental effects of peripheral immune cells on the ischemic brain depend on the time course of stroke. In addition, they summarized the effect of gut microbiota and metabolites on the brain-resident immune cells and raised a question of how the microbiota composition influenced the pathological process of IS.

Besides this, Sarah R. Martha et al. innovatively found that the inflammatory genes and immune mediators in arterial blood distal and proximal to the intracranial thrombus, obtained from patients with emergent large vessel occlusion and who had a mechanical thrombectomy performed, could predict stroke outcomes, such as infarct volume and edema volume, by using machine learning algorithms. Therefore, their research will provide a new thought by using novel methods to diagnose, predict, and design drugs for stroke patients.

In conclusion, this Research Topic collects papers which involve mechanisms of neuroinflammation and neuroimmunology as well as alteration of the peripheral immunity, the bidirectional communication between the injured brain and the immune system, and the possible targeting treatments after IS. However, there are still many undiscovered issues, and one of the battlefields will be on exploring multitargeted immunotherapeutic approaches both protecting the brain and taking the peripheral immune system into consideration, which will bring new light on ischemic stroke treatment.

## Author Contributions

YY wrote the initial draft. XX contributed to reviewing the literature. ZJ submitted the article. LG designed the manuscript and prepared the final version.

## Funding

This study was supported by the National Natural Science Foundation of China (Nos. 82171336 and 81870939 to XX; 82071339 and 81771283 to LG).

## Conflict of Interest

The authors declare that the research was conducted in the absence of any commercial or financial relationships that could be construed as a potential conflict of interest.

## Publisher’s Note

All claims expressed in this article are solely those of the authors and do not necessarily represent those of their affiliated organizations, or those of the publisher, the editors and the reviewers. Any product that may be evaluated in this article, or claim that may be made by its manufacturer, is not guaranteed or endorsed by the publisher.

